# Road traffic noise and children’s inattention

**DOI:** 10.1186/s12940-017-0337-y

**Published:** 2017-11-21

**Authors:** Kjell Vegard Weyde, Norun Hjertager Krog, Bente Oftedal, Per Magnus, Simon Øverland, Stephen Stansfeld, Mark J. Nieuwenhuijsen, Martine Vrijheid, Montserrat de Castro Pascual, Gunn Marit Aasvang

**Affiliations:** 1 Department of Air Pollution and Noise, Norwegian Institute of Public Health, Oslo, Norway; 2 Domain of Health Data and Digitalization, Norwegian Institute of Public Health, Oslo, Norway; 3 Institute of Health and Society, Faculty of Medicine, University of Oslo, Oslo, Norway; 4 Division of Mental Health, Norwegian Institute of Public Health, Bergen, Norway; 5 Faculty of Psychology, University of Bergen, Bergen, Norway; 6 Centre for Psychiatry, Wolfson Institute of Preventive Medicine, Barts and the London School of Medicine, Queen Mary University of London, London, UK; 7 ISGlobal, Centre for Research in Environmental Epidemiology (CREAL), Barcelona, Catalonia Spain; 8 Experimental and Health Sciences, Pompeu Fabra University, 08003 Barcelona, Catalonia Spain; 9 CIBER Epidemiología y Salud Pública (CIBERESP), Madrid, Spain; 10 Pb. 4404 Nydalen, 0403 Oslo, Norway

**Keywords:** Road traffic noise, Inattention, Children, Norwegian mother and child cohort study

## Abstract

**Background:**

An increasing number of children are exposed to road traffic noise levels that may lead to adverse effects on health and daily functioning. Childhood is a period of intense growth and brain maturation, and children may therefore be especially vulnerable to road traffic noise. The objective of the present study was to examine whether road traffic noise was associated with reported inattention symptoms in children, and whether this association was mediated by sleep duration.

**Methods:**

This study was based on the Norwegian Mother and Child Cohort Study conducted by the Norwegian Institute of Public Health. Parental reports of children’s inattention at age 8 were linked to modelled levels of residential road traffic noise. We investigated the association between inattention and noise exposure during pregnancy (*n* = 1934), noise exposure averaged over 5 years (age 3 to 8 years; *n* = 1384) and noise exposure at age 8 years (*n* = 1384), using fractional logit response models. The participants were children from Oslo, Norway.

**Results:**

An association with inattention at age 8 years was found for road traffic noise exposure at age 8 years (coef = .0083, CI = [.0012, .0154]; 1.2% point increase in inattention score per 10 dB increase in noise level), road traffic noise exposure average for the last 5 years (coef = .0090, CI = [.0016, .0164]; 1.3% point increase/10 dB), and for pregnancy road traffic noise exposure for boys (coef = .0091, CI = [.0010, .0171]), but not girls (coef = −.0021, CI = [−.0094, .0053]). Criteria for doing mediation analyses were not fulfilled.

**Conclusion:**

Results indicate that road traffic noise has a negative impact on children’s inattention. We found no mediation by sleep duration.

**Electronic supplementary material:**

The online version of this article (10.1186/s12940-017-0337-y) contains supplementary material, which is available to authorized users.

## Background

About 40% of EU inhabitants are exposed to road traffic noise levels likely to be harmful to health, and the proportion is expected to increase [1, 2]. Observational and experimental studies on adults have shown associations between road traffic noise and sleep disturbance, annoyance, cardiovascular disease [3–6]. Although considered a vulnerable group [6], less research has been done on children. Some studies on noise exposure in children have found increases in blood pressure, stress, annoyance, hyperactivity and behavior difficulties [7–11], and there is some evidence for associations with impaired sleep [12–14]. One of the most robust findings is the association between traffic noise at school and children’s cognition, particularly memory and reading [11, 15]. In addition to findings from cross-sectional studies, associations between traffic noise and cognition have been found in a naturalistic experiment by [16]. When the old Munich airport closed and the new opened, the children near the old airport improved long-term memory, short-term memory and reading, whereas these skills were impaired for children living close to the new airport.

Attention is an important part of cognition, since it determines what information that reaches working memory, where information is evaluated and decisions made [17]. Inattention, as reported by teachers and parents using the criteria for attention-deficit/hyperactivity disorder, has been strongly associated with reading fluency and reading comprehension, writing, mathematics and exam scores [18–20]. In addition, it is associated with increased probability of dropping out of school [21]. It is therefore important to identify factors that can impair attention. Some previous studies have investigated whether traffic noise and inattention are associated. Traffic noise at school has been found to affect attention measured by both neuropsychological tests [15] and teacher observations [22].

Most studies on noise exposure and children’s cognition have focused noise exposure at school, particularly noise from aircraft. Only a few studies have examined the possible impact of road traffic noise at home on children’s cognition. There are different exposure windows in which road traffic noise might impact on inattention, including short-term, long-term or pregnancy periods. First, the effect of road traffic noise might be short-term or instantaneous, for example through its impact on last night’s sleep or by direct interference with communication, increased arousal and annoyance, [3, 23]. The limited literature that exists on noise and children’s sleep suggests a link between increased road traffic noise and sleep disturbances, such as sleep duration, sleep quality and daytime sleepiness [12–14]. Sleep reinvigorates and is important for being alert [24], and impaired sleep has been associated with both behavioral reports and neuropsychological tests of inattention [25–27], although some studies have found no associations [28]. Impaired sleep is also found more often in children with attention deficit/hyperactivity disorder (ADHD), compared to children without ADHD [29]. Studies have indicated that interventions to improve sleep reduce attention deficit symptoms in children with ADHD [30, 31].

Second, long-term impaired sleep causes neuronal loss, impaired brain development and failure to adequately develop coping skills [32, 33], making a longer noise exposure window relevant. In addition, noise exposure and annoyance may cause stress, and it is known that long-term stress in children is associated with several negative health effects, such as impaired brain development and impaired immune system functioning [34, 35]. Among the few studies that have looked at residential noise exposure and inattention, associations have been found with both short-term and long-term noise exposure windows [8, 12]. However, we have only found one study that have investigated whether sleep is a mediating factor in this association: Stansfeld et al. [36] found no mediation by sleep in the association between exposure to aircraft noise and cognitive performance in 9–11 year-old children.

Third, road traffic noise can increase stress [37]. Studies have indicated that maternal stress during pregnancy can impact on children’s development [38]. Thus, road traffic noise during pregnancy is also an exposure window of interest, although the only previous study that has investigated pregnancy noise exposure found no association with behavior reports at age 7 years [8].

In sum, only a few studies have looked into the association between road traffic noise and inattention, and especially studies including residential road traffic noise exposure are lacking. The present study investigated whether road traffic noise was associated with parental-reported inattention in 8-year olds, and whether sleep duration was a mediating factor in this association. We hypothesized that increased road traffic noise level would be associated with increased rating of inattention for all exposure windows, and that this association would be mediated by sleep duration.

## Methods

### Study sample

The study used questionnaire data from the Norwegian Mother and Child Cohort Study, MoBa [39]. MoBa is conducted by the Norwegian Institute of Public Health. It has recruited more than 95,200 women from all over Norway between 1999 and 2008, and 40.6% consented to participate. It includes more than 114,500 pregnancies with biological data and questionnaire data. Mothers received invitations by mail, along with appointments for ultrasound scanning in week 17 or 18 of pregnancy. No exclusion criteria were used in the main study. Three questionnaires were mailed to the mothers during pregnancy, and when the children were 6, 18 and 36 months, and 5, 7 and 8 years. The cohort is described in more detail elsewhere [39].

**Fig. 1:**
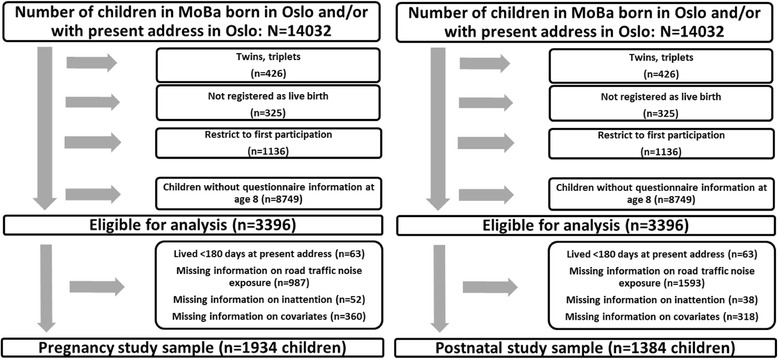
Flow chart of the postnatal (left) and pregnancy (right) study sample selection

MoBa participants with residential address in Oslo were selected because of the availability of noise exposure estimations for this city. All children were born between 2004 and 2007. From an initial sample of 14,032 MoBa participants who at some point had been registered with an Oslo address, we excluded the following: multiple births, births not registered as live birth, all but the oldest participating child of each mother (to avoid multiple dependent observations), and children lacking questionnaire information at age 8. A total of 3396 children met the inclusion criteria. Further, children were excluded if they had lived less than 180 days at the present address (and therefore may not yet had “returned to normal” after the possible stressful life-changing event of moving to a new place), or had missing values on either road traffic noise, sleep duration or any other covariates (see Fig. 1). Two study samples were used: The first sample (pregnancy sample) was constructed to examine noise exposure during pregnancy (*n* = 1934). The second sample (Postnatal sample) was constructed to examine averaged noise exposure over five-years and noise exposure at age 8 (*n* = 1384). There was an overlap between the two samples, with 1029 children present in both. Some children moved out of Oslo before they reached the age of 8 years, and thus were part of the pregnancy sample, but not the postnatal sample. Similarly, some children moved to Oslo after birth, and thus were part of the postnatal sample, but not the pregnancy sample. Another reason why some children were in only one of the samples, was lack of covariate information or children having dropped out of MoBa before age 8.

The analyses are based on MoBa version 9 of the quality-assured data files.

### Noise exposure

Estimations of road and rail traffic noise exposure were conducted by the Agency for Urban Environment, the City of Oslo, in accordance with the Environmental Noise Directive (END) [40]. Noise exposure was modeled using the Nordic Prediction Method [41–43] and the software program CadnaA version 4.3 (DataKustik, GmbH, Germany) [44]. A geographic information system (GIS) approach was used to geocode all the children’s residential addresses as well as the mothers address during pregnancy. Grid predictions of 5 × 5 m^2^ at 4 m height were used to assign noise level to the geocoded addresses. The A-weighted day (07.00–19.00)- evening (19.00–23.00)- and night-time (23.00–07.00) equivalent noise level, L_den_, based on annual average daily traffic (AADT) with diurnal distribution, was employed. L_den_ adds a penalty of 5 dB for the evening and 10 dB for the night. The L_den_ was estimated for the most exposed façade of each child’s residence as well as for the mothers’ residence during pregnancy. Noise exposure from road traffic and rail traffic in the pregnancy sample covered the whole pregnancy period (the exact number of days). In the postnatal sample two exposure windows were employed. The first was road traffic noise exposure at the time of completion of the eight-year questionnaire, L_den_ for the present address and year. The second was the averaged road traffic noise exposure during the five last years before completion of the eight-year questionnaire (1825 days back from the date of completion). The five-year period took into account all addresses occupied during these periods.

Road traffic noise was included in the analyses as continuous variables, whereas rail traffic noise was categorized as unexposed, exposed to less than or equal to L_den_ 30 dB, or exposed to more than L_den_ 30 dB. Children and mothers categorized as unexposed to rail traffic noise had residential address outside a radius of 700 m from a railway line and 300 m for trams and metros, since outside these radii, the rail traffic noise is either nonexistent or is so low that it is masked by other noise sources. The noise exposure assessment was based on input data for the years 2011 and 2006 and included data on topography, building polygons, traffic counts (but estimations for smaller roads without counts), estimated values for 24 h traffic distribution (75% day, 15% evening and 10% night for highways, and 65%, 20% and 15% for municipal roads), signed speed, information on noise barriers, and ground surface (hard or soft). The search radius of 1000 m was used for highways, and 500 m for municipal roads. Residential exposure to rail traffic noise was modeled separately and in a similar way as road traffic noise. For rail traffic, rail time tables were used to obtain information on traffic volume and diurnal distribution of traffic.

### Inattention

Information on inattention was obtained from the 8-year MoBa questionnaire. Mothers were asked to rate their child on nine different inattention items, as part of the Rating Scale for Disruptive Behavior Disorders (RSDBD) [45], corresponding to the nine inattention items of the attention deficit/hyperactivity disorder criteria found in the fourth edition of the Diagnostic and Statistical Manual of Mental Disorders (DSM-IV) [46]. Questions were asked to measure if the child was easily distracted, paid attention to details, could maintain focus over time, often forgot things, disliked activities that demanded attention, had difficulties organizing activities, and so on. Each item was given a score of 0–3 (“never” – “very often”), and a sum score was made (ranging from 0 to 27). The sum score was then made into a fraction by dividing by 27. Cronbach’s Alpha was the same in the pregnancy and postnatal samples: .86.

### Sleep

In the MoBa 8-year-questionnaire, mothers were asked: “Approximately how many hours of sleep per night does your child usually obtain on weekdays?”, with five different response categories: 8 h or less, 9 h, 10 h, 11 h, and 12 h or more. The variable was recoded into three categories: less than 10 h, 10 h, or more than 10 h. Sleep duration was used to assess possible mediation effects.

### Covariates

Covariate information was obtained from the MoBa 8-year questionnaire, the Medical Birth Registry of Norway (MBRN) and Statistics Norway (SSB). Covariates were selected using Directed Acyclic Graphs [35]. DAG is a tool used for covariate selection to minimize the magnitude of bias [47, 48]. Two different DAGs were developed after an extensive literature review, suggesting the covariates’ associations with exposure and outcome. One DAG was made for the analyses with pregnancy noise, and a second was made for the analyses with postnatal noise (see Additional files 1 and 2 in supplementary material). Based on the DAGs, minimal adjustment sets (the minimal selection of variables to be adjusted for in order to avoid a biased result) were suggested using the web based software program dagitty.net [49].

The minimal adjustment set from the DAG for the postnatal sample included gross household income (continuous; postnatal sample: at age 8 years and five-year average) and urbanity (at age 8/five-year average; indicating proximity to city center). An additional set of covariates was considered important to include in the full model because of their well-established association with exposure and outcome, and because they were well measured (few missing values, registry based, etc.). This would increase the chances that potential confounders were included (it is unlikely that the inclusion of only two covariates will account for all confounding). The additional covariates included maternal education (more than 4 years of university/college; less than 4 years of university/college, but more than high school; maximum high school), ethnicity, maternal alcohol consumption during pregnancy (consumed alcohol once a week or more during either of the trimesters), maternal smoking during pregnancy (smoked sometimes or daily during the last trimester), prematurity (gestational length of <259 or > = 259 days) and birth weight (> = vs < 2500 g). Gender and age (months) were also included in the model. Rail traffic noise was categorized as 0 dB, 0–30 dB, or above 30 dB. Air pollution was represented by three components: NO_2_, NO_x_ and PM_2.5_. Air pollution estimates were based on Land Use Regression models generated as part of the ESCAPE and HELIX projects, and are described in more detail elsewhere [50–52]. Exposure windows for the air pollution covariates were the same as for noise. In addition, air pollution during pregnancy was included in the analyses on the postnatal sample. The minimal adjustment set from the DAG for the pregnancy sample included the same covariates as listed above, but covering the pregnancy period only. In both samples, inclusion of rail traffic noise and air pollution was limited to sensitivity analyses. More details on covariates are included in Table 1.

**Table 1:** Characteristics of the study populations

Pregnancy sample				Postnatal sample (5-year data example)			
	<45 dB	45–55 dB	> = 55 dB		<45 dB	45–55 dB	> = 55 dB
N (%)	202 (10.4)	620 (32.1)	1112 (57.5)	N (%)	158 (11.4)	617 (44.6)	587 (44.0)
Mean fraction of inattention score (SD)	.17 (.15)	.19 (.14)	.19 (.15)	Mean fraction of inattention score (SD)	.19 (.16)	.18 (.14)	.20 (.16)
% Boys	47.5	49.4	49.9	% Boys	52.5	50.4	49.8
Age, months (SD)	97.3 (1.2)	97.3 (1.3)	97.5 (1.4)	Age, months (SD)	97.3 (1.2)	97.4 (1.4)	97.5 (1.5)
Income, birth (SD)^a^	825,843	824,736	757,927	Average 5-y income (SD)^a^	1,052,194	1,047,947	920,016
	(585473)	(685930)	(495249)		(585321)	(519580)	(542098)
Mother’s education				Mother’s education			
> 4 years univ./college	35.6	36.3	37.6	> 4 years univ./college	40.5	42.0	41.5
≤ 4 years univ./college	52.5	48.2	50.4	≤ 4 years univ./college	47.5	45.7	44.3
High school	11.9	15.5	12.1	High school	12.0	12.3	14.1
Ethnicity: % western^b^	96.5	93.1	93.5	Ethnicity: % western^b^	92.4	94.3	88.0
Premature^c^, %	4.5	4.2	4.3	Premature^c^, %	8.2	4.1	3.9
Low birth weight^d^, %	1.5	2.9	3.0	Low birth weight^d^, %	3.2	2.9	4.3
Urbanity				Urbanity, birth			
City center	9.9	8.6	34.8	City center			
Between	19.3	27.4	30.0	Between			
Outskirts	70.8	64.0	35.3	Outskirts			
Urbanity,5-y average (SD)				Urbanity, 5-y average (SD)	2.77 (.48)	2.77 (.43)	2.40 (.72)
Maternal alcohol, Pregnancy^e^, % yes	7.9	7.4	7.0	Maternal alcohol, Pregnancy^e^, % yes	7.0	10.2	9.5
Maternal smoking, Pregnancy^f^, % yes	3.5	3.1	4.5	Maternal smoking, Pregnancy^f^, % yes	3.8	3.4	5.1
Sleep duration at age 8				Sleep duration at age 8			
> 10 h				> 10 h	20.9	14.3	18.2
10 h				10 h	62.0	66.6	63.4
< 10 h				< 10 h	17.1	19.1	18.4

^a^Adjusted according to consumer price index. Average of incomes at ages 3, 5, 7 and 8 years, or at birth. Used as a categorical variable in the analyses: <500,000, > = 500,000–800,000, > = 800,000–1,100,000, > = 1,100,000

^b^Dichotomized according to Statistics Norway [53]

^c^Gestational age of less than 259 days

^d^Birth weight of less than 2500 g

^e^Consumed alcohol once a week or more during either of the trimesters

^f^Smoked sometimes or daily during the last trimester

### Statistical analyses

Fractional logit response models [54] were used, since they are shown to be advantageous compared to more traditional linear estimation methods for bounded variables [55]. A crude model (containing road traffic noise, gender and age), a minimal adjustment set model (adding household income and urbanity), and a full model (further adding maternal education, ethnicity, maternal alcohol consumption and smoking during pregnancy, low birth weight and prematurity) were fitted. The modifying effects of gender, income and education were explored, since there is some indication that road traffic noise can affect boys and girls differently [13], and that parents tend to report more inattentive behaviors for boys than girls [56]. Furthermore, children of low socioeconomic status (SES) tend to be exposed to road traffic noise to a greater extent than children from high-SES families [2], and children from relatively high SES groups appear less sensitive to the effect of sleep curtailment on cognitive performance than children from low SES groups [57] The interaction terms were tested with a Wald test, using a significance level of .10 [58, 59]. If significant, analyses were stratified by gender, income or education. To assess whether postnatal noise exposure was more strongly related to inattention than pregnancy noise exposure, or whether the opposite was true, models including both pregnancy and postnatal noise were fitted.

Children of parents who live apart tend to split time between households, but only information on mothers address was available. To address this potential problem, we ran a sensitivity analysis were only children living with both parents were included. We also did sensitivity analyses where we excluded children who, according to the mothers, had been referred to a physician or psychologist due to attentional problems (to account for possible diagnosis of ADHD). Further analyses compared estimates for road traffic noise with and without rail traffic noise included, with and without air pollution, and with and without children born preterm or with a birth weight of less than 2500 g. In only a few cases, not all the items were filled out. For these children, the sum score was divided by the maximum attainable score of those items completed (i.e., 7 completed items would have given a maximum score of 21). These incomplete cases were included in sensitivity analyses.

Adjusted marginal predictions were calculated to aid interpretation of the results. In the analyses stratified on education, fitted values were predicted for different noise categories in each of the education categories, since average marginal predictions were not estimable.

One of the aims was to examine whether sleep duration was a mediator in the noise and inattention association. For a mediation analysis to be done, three criteria need to be fulfilled: There must be an association between the exposure and outcome, and between the exposure and the mediating variable. In addition, the mediation variable must be a statistically significant predictor of the outcome variable in an analysis including both the exposure and the mediating variable [60, 61]. Thus, ordered logistic regression analyses were run to check the association between road traffic noise and sleep duration at age 8 years. A heteroscedastic ordered logistic regression model [62] was used, due to heteroscedasticity caused by the ethnicity covariate. Sleep duration was included as a covariate in the fractional response models with road traffic noise as independent variable an inattention as outcome.

Chi-square analyses and ANOVAs were performed to examine whether the pregnancy and postnatal study samples differed on important covariates from other non-participating MoBa children. The covariates compared included road traffic noise, gender, ethnicity, household income, maternal education, maternal smoking and drinking during pregnancy, and prematurity and low birth weight. Information on the covariates used in these chi-square and ANOVA analyses were obtained from SSB, the MBRN and Health and Welfare Agency, City of Oslo, and was available for all the children, regardless of whether the 8-year questionnaire was completed or not. In order to assess whether results were affected by selection due to loss of participants in MoBa with time, the models were also run using inverse probability weighting (ipw).

All analyses were done in Stata version 14.0 (StataCorp, TX, USA) [63].

## Results

In both samples, there was a decrease in household income with increasing road traffic noise. Apart from these differences, no clear patterns were seen for different categories of road traffic noise (see Table 1). The average road traffic noise level during pregnancy was L_den_ 55.8 dB (SD = 8.3). The average five-year average noise level was L_den_ 54.1 dB (SD = 7.4), and the average level at age 8 was L_den_ 53.1 (SD = 7.8). The correlation between five-year noise and noise at age 8 was high (*r* = .90), whereas pregnancy noise was lower correlated with five-year noise (*r* = .50) and noise at age 8 (*r* = .39). Correlations between noise and air pollution were in the range between .24 and .45 (see Table 2).

**Table 2:** Pearson correlations between road traffic noise and air pollution

	NO2 pregn	NOX pregn	PM2.5 pregn	NO2 5-y	NOX 5-y	PM2.5 5-y	NO2 age 8	NOX age 8	PM2.5 age 8
Pregnancy noise	.43	.45	.44						
5-y average noise	.27	.24	.29	.33	.34	.39			
Noise at age 8	.30	.29	.28				.36	.36	.41

The average rail traffic noise level among those exposed, was L_den_ 41.7 dB in the pregnancy sample (*n* = 1013, SD = 13.1). In the pregnancy sample, five-year rail traffic noise level among those exposed, was L_den_ 39.4 dB (*n* = 855, SD = 11.4), and rail traffic noise at age 8 was L_den_ 39.3 dB (*n* = 739, SD = 11.0). Of all mothers who answered the inattention questions, 98.5 in the pregnancy sample and 98.7% in the postnatal sample responded to all items. The mean inattention score was .19 (SD = .15) in both the postnatal sample and the pregnancy sample. The median was .15 in both samples, and the range 0 to 1. Higher scores were reported for boys than girls (.22 [SD = .16] vs .16 [SD = .13]).

To elucidate potential bias, comparisons between study samples and non-participants were done on several variables. Both study samples had lower road traffic noise exposures, higher maternal education, and higher percentage of children of western origin. Compared to non-participants, the postnatal sample had higher household income and a higher percentage of mothers who reported drinking alcohol during pregnancy. Otherwise, the study samples did not differ from non-participants (see Table 3).

**Table 3:** Comparisons of study samples and non-participants

	Pregnancy	sample		Postnatal	sample	
	Study sample	Non-participants	*p*-value	Study sample	Non-participants	*p*-value
Average 5-y road traffic noise (SD), n				54.1 (7.4), *n* = 1384	55.1 (7.6), *n* = 4834	<.0001
Road traffic noise at age 8 (SD), n				53.1 (7.8), *n* = 1384	53.9 (8.2), *n* = 5484	.001
Road traffic noise, pregnancy (SD), n	55.8 (8.3), n = 1934	56.6 (8.4), *n* = 6425	.0001			
Average 5-y income (SD), n				992,139 (540759), *n* = 1384	901,693 (580480) *n* = 7579	<.0001
Income at age 8 (SD), n				1,046,052 (621477), *n* = 1384	948,394 (678449) *n* = 7719	<.0001
Income, pregnancy (SD), n	786,438 (573046), *n* = 1934	767,439 (587234), *n* = 9688	.19			
Mother’s education, n	*n* = 1934	*n* = 10,036		*n* = 1384	*n* = 10,586	
> 4 years univ./college	37.0	33.5	<.001	41.6	33.1	<.001
≤ 4 years univ./college	49.9	47.1		45.3	47.8	
High school	13.1	19.4		13.1	19.1	
Ethnicity, % western, n	93.7, *n* = 1934	86.6, *n* = 10,148	<.001	91.3, *n* = 1384	87.3, *n* = 10,698	<.001
Gender, % boys, n	49.5, *n* = 1934	50.9, *n* = 10,206	.26	50.4, *n* = 1384	50.7, *n* = 10,756	.81
Maternal smoking, pregnancy, % yes	3.9, *n* = 1934	4.8n *n* = 8871	.12	4.2, *n* = 1384	4.7, *n* = 9421	.44
Maternal alcohol consumption, pregnancy, % yes	7.2, *n* = 1934	7.3, *n* = 7278	.98	9.5, *n* = 1384	6.9, *n* = 7828	<.001
% birth weight < 2500 g, n	2.8, *n* = 1934	3.2, *n* = 10,202	.33	3.5, *n* = 1384	3.1, *n* = 10,752	.37
% premature, n	4.3, *n* = 1934	4.7, *n* = 10,192	.39	4.5, *n* = 1384	4.7, *n* = 10,742	.73

**Fig. 2:**
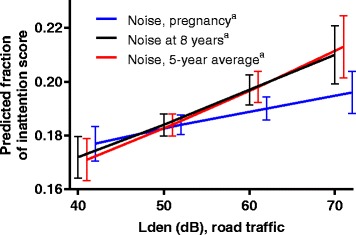
Graphs displaying the relationship between different exposure windows of road traffic noise and inattention. ^a^Adjusted for: road traffic noise, age, gender, household income, maternal education, urbanity, ethnicity, maternal alcohol consumption during pregnancy, maternal smoking during pregnancy, low birth weight (>=/<2500 g) and prematurity (>=/<259 days)

No association was found between pregnancy road traffic noise and inattention at age 8 years for the full pregnancy sample (coef = .0042, CI = [−.0013, .0096]; see Fig. 2 and Table 4).

**Table 4:** Main results showing effect estimates for road traffic noise with 95% CIs

		Pregnancy	sample	Postnatal	sample
		Pregnancy (*n* = 1934)		Noise at 8 years (*n* = 1384)	5-year average (3–8 y; *n* = 1384)
Analysis	N	Coeff. (95% CI)	N	Coeff. (95% CI)	Coeff. (95% CI)
Crude^a^:	1934	.0033 (−.0019, .0084)	1384	.0088 (.0019, .0157)	.0100 (.0029, .0171)
Minimal adjustment set^b^:	1934	.0043 (−.0011, .0097)	1384	.0084 (.0013, .0154)	.0094 (.0021, .0168)
Main^c^:	1934	.0042 (−.0013, .0096)	1384	.0083 (.0012, .0154)	.0090 (.0016, .0164)
Stratified by maternal education:					
High school			181	−.0079 (−.0267,.0109)	−.0091 (−.0289,.0107)
Up to 4 years of university/college			627	.0064 (−.0035, .0163)	.0059 (−.0048, .0166)
5 or more years of university/college			576	.0179 (.0061, .0297).	.0218 (.0100, .0337)
Stratified by household income:					
< 600,000 NOK	605	.0057 (−.0039, .0153)			
600,000–900,000 NOK	899	.0017 (−.0063, .0097)			
> = 900,000 NOK	430	.0087 (−.0041, .0215)			
With rail traffic noise (*n* = 1384/1934):	1934	.0040 (−.0015, .0096)	1384	.0083 (.0011, .0154)	.0085 (.0010, .0159)
Without referred (*n* = 1352/1889):	1889	.0046 (−.0007, .0098)	1352	.0071 (.0004, .0137)	.0082 (.0012, .0151)
Without parents living apart (*n* = 1208/1722):			1208	.0074 (−.0004, .0152)	.0075 (−.0007, .0165)
Without premature (*n* = 1322/1851):	1851	.0052 (−.0005, .0109)	1322	.0099 (.0027, .0170)	.0112 (.0037, .0186)
Without low birth wgt (*n* = 1335/1880):	1880	.0049 (−.0007, .0105)	1335	.0081 (.0009, .0153)	.0093 (.0019, .0168)
Without airpoll at 8 years (*n* = 401):			401	.0091 (−.0042, .0224)	
With NO2 at 8 years (*n* = 401):			401	.0077 (−.0061, .0215)	
With NOX at 8 years (*n* = 401):			401	.0079 (−.0057, .0214)	
With PM2.5 at 8 years (*n* = 401):			401	.0093 (−.0044, .0229)	
Without 5-y average air pollution (*n* = 369):			369		.0054 (−.0084, .0191)
With NO2, 5-y average (*n* = 369):			369		.0042 (−.0096, .0181)
With NOX, 5-y average (*n* = 369):			369		.0040 (−.0097, .0176)
With PM2.5, 5-y average (*n* = 369):			369		.0041 (−.0097, .0180)
Without air poll, pregnancy (*n* = 1314/1931):	1931	.0042 (−.0013, .0097)	1314	.0081 (.0010, .0152)	.0091 (.0016, .0165)
With NO2, pregnancy (*n* = 1314/1931):	1931	.0039 (−.0020, .0098)	1314	.0074 (.0001, .0147)	.0083 (.0006, .0160)
With NOX, pregnancy (*n* = 1314/1931):	1931	.0047 (−.0012, .0107)	1314	.0075 (.0003, .0147)	.0084 (.0007, .0160)
With PM2.5, pregnancy (*n* = 1314/1931):	1931	.0035 (−.0023, .0093)	1314	.0078 (.0005, .0150)	.0088 (.0011, .0164)

^a^Covariates included in crude model: age and gender

^b^Covarites incuded in the minimal adjustment set model: age, gender, income and urbanity

^c^Covariates included in main model: road traffic noise, age, gender, household income, maternal education, urbanity, ethnicity, maternal alcohol consumption during pregnancy, maternal smoking during pregnancy, low birth weight (>=/<2500 g) and prematurity (>=/<259 days)

There was a statistically significant effect modification by gender (*p* = .05). Stratifying by gender revealed an association between road traffic noise and inattention for boys (*n* = 957, coef = .0091, CI = [.0010, .0171], with marginal predictions of .184 at L_den_ 40 dB and .212 at L_den_ 60 dB, corresponding to an inattention score of 5.0 and 5.7, respectively), but not girls (*n* = 977, coef = −.0021, CI = [−.0094, .0053]). There was also a statistically significant effect modification by income (*p* = .03). Stratifying by income, a positive tendency was found between noise and inattention for all levels of income, with the strongest tendency for the highest level (see Table 4). Marginal predictions for the lowest income categories were, for L_den_ 45 dB, 55 dB, and 65 dB: .19 (CI = [.17, .21]), .20 (CI = [.19, .21]), and .21 (CI = [.19, .23]). For the middle income category, marginal predictions were: .18 (CI = [.17, .20]), .18 (CI = [.17, .19]), and .19 (CI = [.17, .20]); and for the highest income category: .16 (CI = [.14, .18]), .17 (CI = [.16, .18]), and .18 (CI = [.16, .20]). There was no effect modification by education (*p* = .36).

Including rail traffic noise or children with incomplete inattention score, or excluding children not living with both parents, excluding children referred to a physician due to attention problems, excluding premature children, or excluding children with a birth weight of less than 2500 g did not change the estimates much. The effect estimates were somewhat lowered when including air pollution covariates. (See Table 4.)

An association was found between road traffic noise exposure at age 8 years and the fractioned inattention score (coef = .0083, CI = [.0012, .0154]). The average marginal effects showed that this corresponded to an increase in inattention score of about 1.2 percentage points per L_den_ 10 dB increase in road traffic noise exposure. For example, at L_den_ 40 dB, the average inattention fraction was .172, whereas it was .197 at L_den_ 60 dB. That corresponded to 17.2 and 19.7% of the maximum inattention score, or an inattention score of 4.6 and 5.3, respectively. Using the five-year road traffic noise exposure gave similar noise estimates (coef = .0090, CI = [.0016, .0164], with marginal predictions of .169 at L_den_ 40 dB and .196 at L_den_ 60 dB, corresponding to an inattention score of 4.6 and 5.3, or an increase of about 1.3 percentage points in inattention score per 10 dB increase in road traffic noise exposure). (See Fig. 2 and Table 4.)

There was a statistically significant effect modification by education in both analysis with noise at age 8 (*p* = .06), and analysis with five-year noise (*p* = .01). When stratifying by education, positive associations were revealed for both exposure windows between noise and inattention in children of highly educated mothers. A similar tendency was seen for the middle education category, whereas an opposite tendency was found for children of less educated mothers (see Table 4). For children of less educated mothers, compared to children of more educated mothers, fitted values were higher for the less exposed (at L_den_ < 50 dB: .24 vs .17 for noise at age 8; .25 vs .16 for five-year noise), but similar for the higher exposed (at L_den_ > =60 dB: .21 vs .21 for noise at age 8; .21 vs .21 for five-year noise). There was no effect modification by gender for either noise at age 8 (*p* = .99) or five-year noise (*p* = .93), or by income (noise at age 8: *p* = .63; five-year noise: *p* = .43).

Including rail traffic noise, excluding children not living with both parents, excluding children referred to a physician due to attention problems, excluding premature children, or excluding children with a birth weight of less than 2500 g did not change the estimates much (see Table 4). The effect estimates were somewhat lowered when including air pollution covariates (see Table 4).

Including noise at age 8 in the pregnancy main model reduced estimates for pregnancy noise by 52.4%, whereas 5-year average noise reduced estimates for pregnancy noise by 73.8%. Including pregnancy noise in the model with noise at age 8 reduced the effect estimates by 5.6%, whereas including pregnancy noise in the model with 5-year average noise reduced the estimates by 2.6% (see Table 5).

**Table 5:** Results of analysis including both postnatal and pregnancy road traffic noise^a^

Analysis	Pregnancy noise	Analysis	Noise at age 8	5-year average noise
			Coeff. (95% CI)	
	Coeff. (95% CI)			
				Coeff. (95% CI)
Main^b^ (*n* = 1029):	.0042 (−.0030, .0114)	Main^b^ (*n* = 1029):	.0071 (−.0007, .0149)	.0078 (−.0004, .0159)
Noise at 8 years as covariate (*n* = 1029):	.0020 (−.0055, .0096)	Pregnancy noise as covariate, full (*n* = 1029):	.0067 (−.0016, .0149)	.0076 (−.0013, .0166)
5-year average noise as covariate (*n* = 1029):	.0011 (−.0066, .0088)			

^a^Pregnancy noise included as a covariate in the postnatal sample analyses, and postnatal noise included in the pregnancy sample analyses

^b^Including only children who are part of both pregnancy and postnatal samples. Covariates included are road traffic noise, age, gender, household income, maternal education, urbanity, ethnicity, maternal alcohol consumption during pregnancy, maternal smoking during pregnancy, low birth weight (>=/<2500 g) and prematurity (>=/<259 days)

Criteria for performing a mediation analysis were not met with the sleep variable, since road traffic noise was not associated with sleep duration at age 8. Sleep duration was associated with inattention, however, with reduced sleep duration associated with increased inattention (results not shown).

Analyses with inverse probability weights (see Additional file 3: Table S1) gave lower effect estimates for road traffic noise than the original analyses. In the ipw analyses on the pregnancy sample, Wald tests of interaction terms were statistically significant for gender (*p* = .07), but not for income (*p* = .19) and education (*p* = .81). In the postnatal sample, Wald tests of interactions revealed similar results to those in the postnatal sample for both gender (noise at age 8: *p* = .84; five-year noise: *p* = .90), income (noise at age 8: *p* = .61; five-year noise: *p* = .43) and education (noise at age 8: *p* = .03; five-year noise: *p* = .01).

In sensitivity analyses with ipw, the effect estimates for road traffic noise were lower, but the pattern of change was the same as in the original analyses (see Additional file 3: Table S1).

In analyses with ipw, including pregnancy noise in the models with noise at age 8 or five-year noise, reduced the effect estimates for noise by 11.7 and 10.9%, respectively. Including noise at age 8 or five-year noise in the model with pregnancy noise, reduced the effect estimates by 36.1 and 66.7%, respectively (see Additional file 3: Table S2).

## Discussion

In the present study, inattention at 8 years was associated with both road traffic noise exposure at 8 years and five-year noise exposure (from age 3 to 8 years). An effect modification by education was found, with a positive effect estimate for children of highly educated mothers, and a negative effect estimate for children of less educated mothers. No statistically significant association was found between pregnancy noise and inattention in the full pregnancy sample. However, gender modified the association, revealing an association for boys, but not girls. Effect modification was also found with household income in the original analysis, but not in the analyses with ipw, questioning this finding. Sleep duration was associated with inattention, but criteria for doing mediation analyses were not met, since road traffic noise was not associated with sleep duration.

The findings of positive associations between postnatal road traffic noise and inattentiveness, and the lack of association between pregnancy noise and inattention in the full pregnancy sample, are in line with previous studies [8, 12, 22]. However, the effect modification by gender and education in the inattention study is in contrast to Hjortebjerg et al. [8], who found no modification. As seen in the stratified results, children of less educated mothers scored higher on inattention, as expected. Previous studies have found beneficial effects of noise on cognitive performance among children rated by teachers as being sub-attentive, but impairing effects of noise on children rated as super-attentive [64, 65]. This could be one explanation why effects of road traffic noise were cancelled out, or slightly reversed, in the low education category. The association with pregnancy noise for boys aligns with results from previous studies where prenatal stress affected boys and girls differently, and with previous reports of ADHD symptoms, where effects are more pronounced in boys [66, 67]. The analyses with both pregnancy and postnatal noise exposure included in the models resulted in large reductions in the pregnancy noise estimate for the full sample, but almost no reduction in the postnatal noise estimates, suggesting that postnatal noise exposure is more closely related to inattention at age 8 years. These findings were confirmed in the ipw analyses.

A difference between our study and those of Hjortebjerg et al. [8] and Tiesler et al. [12], is that those studies used the Strength and Difficulties Questionnaire (SDQ) [68] for assessing inattention. The use of different instruments may explain the different findings regarding effect modification.

SDQ has fewer and somewhat different inattention items compared to the two scales mentioned below. Forns et al. [22] used teacher ratings based on the ADHD Rating Scale-IV [69], and the present study used RSDBD [45]. Arriving at similar conclusions about the relationship between children’s exposure to road traffic noise and inattention using different instruments and different kinds of reporters (mothers and teachers) strengthens the findings.

Sleep duration was associated with inattention, as expected. Despite that, criteria for doing mediation analyses were not met, since road traffic noise was not associated with sleep duration. This was a little surprising, since an association between noise and sleep duration was found for 7 year-old girls in a recent study, using an overlapping population to that in the present study [13]. One reason for this discrepancy could be the use of different covariate information and only half as many participants (that study had *n* = 2665). The sleep duration variable was a relatively crude measure of sleep. It is possible that we would have found mediation by sleep duration using a more detailed measure. Other aspects of sleep might mediate the noise and inattention association, such as low sleep efficiency or alterations of sleep stages.

We did not have the information to examine other sleep factors in the present study. Our findings are nevertheless in line with a study by Stansfeld et al. [36] that did not find any mediation by sleep in the association between aircraft noise and cognitive performance in 9–11 year-old children.

There is a possibility that the observed associations between road traffic noise and inattention were due to exposure during the wake time, rather than during sleep. For example, noise might have directly interfered with communication, caused annoyance, or affected inattention through other factors [3], that in turn have affected the observed attentiveness of the child. Unfortunately, we did not have enough information to explore this further.

When including air pollution, NO_2_ and NO_X_, the effect estimates for road traffic noise were somewhat decreased (see Table 4), suggesting that these air pollution components to some extent confounded the association between road traffic noise and inattention. This was a little different from the findings obtained by Hjortebjerg et al. [8] and Forns et al. [22], who found no confounding by air pollution. In the other sensitivity analyses, no big changes in the effect estimates for road traffic noise were seen when including rail traffic noise or excluding those referred due to attention problems, or those not living with both parents. Excluding those who had a birth weight of less than 2500 g, or those who were born pre-term, increased the effect estimates for road traffic noise.

Being born premature or with a low birth weight showed a tendency towards increased inattention symptoms (results not shown). Thus, the explanation regarding the effect modification by education, might also be used to explain the results of these sensitivity analyses.

The present study has several strengths. First, the study sample had a large variance in noise exposure, strengthening the opportunity to detect associations. Modelled noise exposure was based on all historic addresses occupied by the children. Also, through sensitivity analyses, accounting for the fact that some children likely switched between addresses, since they had parents who lived apart, increased the precision of the exposure. Second, covariates were selected with the aid of the DAG framework, reducing the chances of obtaining a biased result [70]. Several covariates were obtained from the MBRN and SSB, with minimal missing data and high accuracy.

The study also has some limitations. First, we did not have information on road traffic noise exposure at school, which might have confounded our results. Another limitation relates to the use of parental ratings of inattention. Ideally, these should have been combined with reports from teachers, but such reports were not accessible in our study. However, as mentioned, Forns et al. [22] found associations between road traffic noise and teacher-reported inattention, indicating that the association can be observed through such measurement models. In addition, an advantage with subjective reports over neuropsychological tests of attention, is that inattention problems are not always detected with tests, but better observed through actual behavior [26].

Whether people sleep with windows opened or closed, affects the actual road traffic noise exposure [14], as do location of bedrooms (towards most or least exposed façade). For example, the actual noise levels inside the bedrooms may be more similar between children with high and low outdoor levels, due to the noise attenuation effect of closed windows. Lack of such detailed information could increase measurement error and reduce the estimates [14, 71].

The differences between the study sample and the non-study sample were, although statistically significantly different on several covariates (see Table 3), generally small. Income and education differences in the postnatal sample and education differences in the pregnancy sample were greater. However, income and education did not change the estimates much when left out of the models (at most 7%). Still, income at birth, education and ethnicity were used as predictors of whether or not children were included in the analyses, and predictions from those analyses were used to compute inverse probability weights. The analyses with ipw showed that caution should be taken when reporting the effect estimates of the present study, since some influence of selection bias were indicated. The effect estimates reported in the original analyses might have been overestimated. Still, conclusions based on the ipw analyses were the same as those based on the original analyses.

In a large Norwegian study cohort with reports from parents and teachers, lower prevalence of ADHD among participants than among non-participants was found [72]. Based on that study, it seems likely that highly inattentive children were underrepresented in our study. Such an underrepresentation might have led to overestimation of effects, as the ipw analyses indicated, possibly since children high in ADHD symptoms seem to be less negatively affected by noise [64, 65].

## Conclusions

Results indicate that exposure to road traffic noise has a negative impact on children’s functioning. Future studies should employ a longitudinal design and further assess the mediating role of sleep, using several and more detailed measures of sleep. The findings underline the importance of protecting children against road traffic noise.

## Additional files

Additional file 1: Directed acyclic graphs for pregnancy sample. (PDF 304 kb)

Additional file 2: Directed acyclic graphs for postnatal sample. (PDF 306 kb)

Additional file 3: Table S1. Main results of ipw analyses, showing effect estimates for road traffic noise with 95% CIs. **Table S2.** Results of weighted analysis including both postnatal and pregnancy road traffic noise^a^. (DOCX 56 kb)

## Abbreviations

END: Environmental Noise Directive DIRECTIVE 2002/49/EC OF THE EUROPEAN PARLIAMENT AND OF THE COUNCIL of 25 June 2002
L_den_: The A-weighted day (07.00–19.00)- evening (19.00–23.00)- and night-time (23.00–07.00) equivalent noise level
MBRN: Medical Birth Registry of Norway
MoBa: Norwegian Mother and Child Cohort Study
SES: Socioeconomic status
SSB: Statistics Norway
