# Supplementary material for: Road traffic noise and children’s inattention
Source: Environ Health. 2017 Nov 21;16:127. doi: 10.1186/s12940-017-0337-y (PMC5698983; doi:10.1186/s12940-017-0337-y)

Additional File 2

Road traffic noise and children's inattention

Directed acyclic graphs for postnatal sample

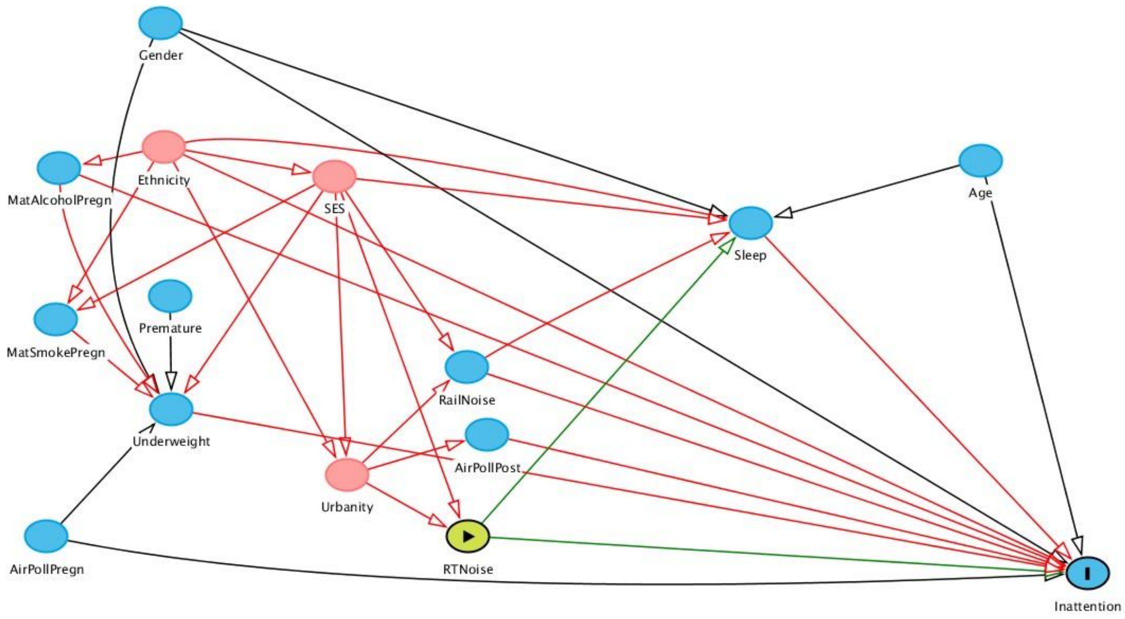

Supplement: Supplementary file 2 — Directed acyclic graphs for postnatal sample. (PDF 306 kb) [file 12940_2017_337_MOESM2_ESM.pdf]
