# Supplementary material for: Road traffic noise and children’s inattention
Source: Environ Health. 2017 Nov 21;16:127. doi: 10.1186/s12940-017-0337-y (PMC5698983; doi:10.1186/s12940-017-0337-y)
Supplement: Supplementary file 3 — Main results of ipw analyses, showing effect estimates for road traffic noise with 95% CIs. Table S2. Results of weighted analysis including both postnatal and pregnancy road traffic noisea. (DOCX 56 kb) [file 12940_2017_337_MOESM3_ESM.docx]

#### *Table S1: Main results of ipw analyses, showing effect estimates for road traffic noise with 95% CIs.*

|  |  | Pregnancy sample |  | Postnatal | sample |
| --- | --- | --- | --- | --- | --- |
|  |  | Pregnancy  (n=1934) |  | Noise at 8 years  (n=1384) | 5-year average  (3-8 y; n=1384) |
| Analysis | N | Coeff. (95% CI) | N | Coeff. (95% CI) | Coeff. (95% CI) |
| Main^a^: | 1934 | .0038 (-.0020, .0097) | 1384 | .0065 (-.0009, .0138) | .0070 (-.0007, .0147) |
| With rail traffic noise: | 1934 | .0038 (-.0021, .0096) | 1384 | .0065 (-.0009, .0139) | .0066 (-.0012, .0143) |
| Without referred: | 1889 | .0048 (-.0006, .0102) | 1352 | .0055 (-.0012, .0122) | .0065 (-.0005, .0135) |
| Without parents living apart: | 1722 | .0030 (-.0033, .0093) | 1208 | .0053 (-.0029, .0136) | .0055 (-.0031, .0142) |
| Without premature: | 1851 | .0054 (-.0006, .0114) | 1322 | .0088 (.0014, .0162) | .0100 (.0023, .0177) |
| Without low birth wgt: | 1880 | .0050 (-.0009, .0110) | 1335 | .0069 (-.0005, .0143) | .0081 (.0004, .0158) |
| Stratified by maternal education: |  |  |  |  |  |
| High school |  |  | 181 | -.0075 (-.0267,.0116) | -.0095 (-.0296,.0106) |
| Up to 4 years of university/college |  |  | 627 | .0059 (-.0044, .0161) | .0053 (-.0057, .0164) |
| 5 or more years of university/college |  |  | 576 | .0180 (.0059, .0301). | .0226 (.0109, .0343) |
| Stratified by household income: |  |  |  |  |  |
| <600000 NOK | 605 | .0083 (-.0019, .0186) |  |  |  |
| 600000-900000 NOK | 899 | -.0009 (-.0096, .0077) |  |  |  |
| >= 900000 NOK | 430 | .0094 (-.0033, .0222) |  |  |  |
| Without airpoll at 8 years: |  |  | 401 | .0066 (-.0080, .0212) |  |
| With NO2 at 8 years: |  |  | 401 | .0048 (-.0104, .0200) |  |
| With NOX at 8 years: |  |  | 401 | .0051 (-.0097, .0200) |  |
| With PM2.5 at 8 years: |  |  | 401 | .0065 (-.0084, .0214) |  |
| Without 5-y average air pollution: |  |  | 369 |  | .0045 (-.0102, .0193) |
| With NO2, 5-y average: |  |  | 369 |  | .0028 (-.0123, .0179) |
| With NOX, 5-y average: |  |  | 369 |  | .0030 (-.0118, .0177) |
| With PM2.5, 5-y average: |  |  | 369 |  | .0023 (-.0126, .0172) |
| Without air poll, pregnancy: | 1931 | .0039 (-.0019, .0097) | 1314 | .0065 (-.0010, .0140) | .0072 (-.0007, .0151) |
| With NO2, pregnancy: | 1931 | .0032 (-.0029, .0094) | 1314 | .0055 (-.0021, .0131) | .0060 (-.0021, .0141) |
| With NOX, pregnancy: | 1931 | .0042 (-.0021, .0105) | 1314 | .0057 (-.0018, .0132) | .0062 (-.0018, .0142) |
| With PM2.5, pregnancy: | 1931 | .0029 (-.0032, .0091) | 1314 | .0060 (-.0017, .0136) | .0066 (-.0015, .0147) |

^a^Covariates included in main model: road traffic noise, age, gender, household income, maternal education,

urbanity, ethnicity, maternal alcohol consumption during pregnancy, maternal smoking during pregnancy,

low birth weight (>=/<2500 g) and prematurity (>=/<259 days).

#### Table S2: Results of weighted analysis including both postnatal and pregnancy road traffic noise^a^

| Analysis | Pregnancy noise  Coeff. (95% CI) | Analysis | Noise at age 8  Coeff. (95% CI) | 5-year average noise  Coeff. (95% CI) |
| --- | --- | --- | --- | --- |
| Main^b^ (n=1029): | .0036 (-.0045, .0117) | Main^b^ (n=1029): | .0060 (-.0021,.0141) | .0064 (-.0021, .0148) |
| Noise at 8 years as covariate (n=1029): | .0023 (-.0060, .0107) | Pregnancy noise as covariate, full (n=1029): | .0053 (-.0033, .0140) | .0057 (-.0037, .0151) |
| 5-year average noise as covariate (n=1029): | .0012 (-.0074, .0098) |  |  |  |

^a^Pregnancy noise included as a covariate in the postnatal sample analyses, and postnatal noise included in the pregnancy sample analyses.

^b^Including only children who are part of both pregnancy and postnatal samples. Covariates included are road traffic noise, age, gender, household income, maternal education, urbanity, ethnicity, maternal alcohol consumption during pregnancy, maternal smoking during pregnancy, low birth weight (>=/<2500 g) and prematurity (>=/<259 days).
